# Probiotic Potential of *Lactobacillus* Strains with Antimicrobial Activity against Some Human Pathogenic Strains

**DOI:** 10.1155/2014/927268

**Published:** 2014-07-03

**Authors:** Parisa Shokryazdan, Chin Chin Sieo, Ramasamy Kalavathy, Juan Boo Liang, Noorjahan Banu Alitheen, Mohammad Faseleh Jahromi, Yin Wan Ho

**Affiliations:** ^1^Institute of Bioscience, Universiti Putra Malaysia (UPM), 43400 Serdang, Selangor, Malaysia; ^2^Faculty of Biotechnology and Biomolecular Sciences, Universiti Putra Malaysia (UPM), 43400 Serdang, Selangor, Malaysia; ^3^Faculty of Pharmacy, Universiti Teknologi MARA, 42300 Puncak Alam, Selangor, Malaysia; ^4^Institute of Tropical Agriculture, Universiti Putra Malaysia (UPM), 43400 Serdang, Selangor, Malaysia

## Abstract

The objective of this study was to isolate, identify, and characterize some lactic acid bacterial strains from human milk, infant feces, and fermented grapes and dates, as potential probiotics with antimicrobial activity against some human pathogenic strains. One hundred and forty bacterial strains were isolated and, after initial identification and a preliminary screening for acid and bile tolerance, nine of the best isolates were selected and further identified using 16 S rRNA gene sequences. The nine selected isolates were then characterized *in vitro* for their probiotic characteristics and their antimicrobial activities against some human pathogens. Results showed that all nine isolates belonged to the genus *Lactobacillus*. They were able to tolerate pH 3 for 3 h, 0.3% bile salts for 4 h, and 1.9 mg/mL pancreatic enzymes for 3 h. They exhibited good ability to attach to intestinal epithelial cells and were not resistant to the tested antibiotics. They also showed good antimicrobial activities against the tested pathogenic strains of humans, and most of them exhibited stronger antimicrobial activity than the reference strain *L. casei* Shirota. Thus, the nine *Lactobacillus* strains could be considered as potential antimicrobial probiotic strains against human pathogens and should be further studied for their human health benefits.

## 1. Introduction

Probiotics are defined as “live microorganisms which when administered in adequate amounts confer a health benefit on the host” [1]. During the last decade, the use of probiotics for human has received increasing attention as scientific evidence continues to accumulate on the properties, functionality, and beneficial effects of probiotic bacteria on humans. The search for more new probiotics is driven by the growing demand for probiotic functional food and beverages and dietary supplements due to rising levels of health consciousness and growing consumer awareness regarding gut health and the concept of preventive health care. It is now well established that some of the infections and disorders in the human body, such as irritable bowel syndrome, inflammatory bowel disease, and antibiotic-induced diarrhea, could be due to deficient or compromised intestinal microflora, and probiotics have been considered to be one of the disease control strategies to overcome such disorders [2]. Thus, probiotics have become increasingly considered for use in the food industry. Lactic acid bacteria, especially* Lactobacillus*, are the most commonly used microorganisms as probiotics because of the perception that they are desirable members of the intestinal microflora and because these bacteria have “Generally Recognized As Safe” (GRAS) status. The growing interest in probiotics has resulted in many purported probiotic products being marketed without adequate studies on the probiotic properties of the strains leading to problems of inconsistent efficacy of the products. Since the properties of probiotic are strain-specific, the quality of products is closely linked to the individual strains in the products. Thus, they should be correctly identified, and their probiotic properties properly studied. However, several studies have reported misidentification or mislabeling of probiotic species or presence of unspecified species in many commercial probiotic products [3–7]. The guidelines proposed by FAO/WHO [8] for evaluation of probiotics recommended that every potential probiotic strain should be correctly identified using both phenotypic and genotypic methods, followed by various tests to investigate its survival ability and functional properties. Acidity, presence of bile salts, and pancreatic enzymes in the gastrointestinal tract (GIT) are some of the major stresses that an orally taken probiotic encountered in the GIT. It is essential that a potential probiotic strain is able to tolerate these stress conditions in order to survive in the GIT. Apart from being able to survive, a probiotic strain also has to be able to adhere to and subsequently colonize (at least temporarily) the intestinal tract. Since the GIT is a dynamic environment, the flow of the gut digesta may wash out any bacterium not attached to the intestinal mucosa. Thus, probiotic strains with adherent ability are more likely to have an increased opportunity to colonize the GIT. The transmission of antibiotic resistance genes from food bacteria to commensal or pathogenic bacteria in the gut is a major health concern. In recognition of the importance of assuring safety, the FAO/WHO [8] guidelines recommended that the antibiotic resistance/susceptibility pattern of every probiotic strain (including bacteria with GRAS status) be determined.

Recent concerns on the rampant and indiscriminate use of antibiotics for disease treatments and growth promotion of livestock and the development of antibiotic resistant pathogens have led to increased interest in the application of probiotics and their antimicrobial metabolites as alternative antimicrobial strategies for treatment and prevention of infections. Hence, antimicrobial activity against pathogens is a desirable property of a potential probiotic strain.

The present study was carried out to isolate, identify, and characterize some lactic acid bacteria from human milk, infant feces, and fermented grapes and dates, as potential probiotics with antimicrobial activity against microorganisms that are pathogenic to humans. The probiotic properties were investigated through* in vitro *assays.

## 2. Materials and Methods

### 2.1. Isolation of Lactic Acid Bacteria

Samples of breast milk were collected aseptically from five healthy women, within four months of given birth to healthy babies, in Kuala Lumpur, Malaysia. The nipple and mammary areola of the breast were wiped with 70% ethanol and about 5 mL of milk was collected in a sterile test tube using a sterile breast pump. Samples of infant feces were collected in sterile test tubes from five healthy breast-fed infants of one to four months old, in Kuala Lumpur, Malaysia. Informed consents were obtained from the donors of breast milk and mothers of the infants. Samples of fermented grapes and dates were obtained from Iranian fermented grapes and dates which are commonly used as condiments to flavor or complement foods. The grapes and dates (500 g each) were fermented in 100 mL of water and 20 g NaCl in 1 L bottles for 40 days at room temperature. Samples were taken from five bottles of fermented grapes and five bottles of fermented dates. Each bottle was shaken by hand for 30 s and left to stand for 5 min at room temperature, after which the supernatant was collected.

Tenfold serial dilutions of 10^−8^ to 10^−10^ of the samples were prepared using 0.5% peptone water (Sigma, USA). From each dilution, 100 *μ*L was spread-plated on de Man, Rogosa and Sharpe (MRS) agar medium (Merck, Germany) and incubated for 72 h at 37°C in anaerobic jars (Oxoid, UK) containing gaspack (AnaeroGen, Oxoid, UK) (oxygen level <1%, CO_2_ level between 9 and 13%). After the incubation period, colonies were randomly picked from the plates and subcultured three times on fresh MRS agar plates. The cultures were kept in MRS broth containing 20% (v/v) glycerol at −80°C.

One hundred and forty bacterial strains were isolated from human milk, infant feces, fermented dates, and fermented grapes. Of the 140 isolates, 42 were from fermented dates, 30 from fermented grapes, 32 from breast milk, and 36 from infant feces. Gram staining and catalase test on the isolates showed that, out of 140 isolates, 94 isolates were gram positive and catalase negative, indicating that they were probably lactic acid bacteria (LAB). A rapid preliminary screening for acid and bile tolerance using turbidity (OD at 620 nm) as a growth measurement was then carried out on these isolates. For acid tolerance, 1% (v/v) of overnight culture (7 to 8 log CFU/mL) was inoculated into phosphate buffer saline (PBS) (8 g NaCl, 0.2 g KCl, 1.44 g Na_2_HPO_4_, and 0.24 g KH_2_PO_4_ in 1 L distilled water) at pH 7.2 (control) and pH 3 (adjusted with 1 N HCL) (acidic condition). The culture was then incubated at 37°C for 3 h, after which 1% (v/v) of cell suspension was inoculated into 10 mL of MRS broth and incubated at 37°C for 24 h. Cell growth was assessed by measuring optical density (OD) at 620 nm. For the bile tolerance, 1% (v/v) of overnight culture (7 to 8 log CFU/mL) was inoculated into 10 mL of MRS broth with or without (control) 0.3% (w/v) oxgall (Sigma, USA) and incubated at 37°C for 4 h, after which growth was assessed by measuring OD at 620 nm. From this preliminary screening, three isolates from human milk, three isolates from infant feces, one isolate from fermented grapes, and two isolates from fermented dates, which exhibited growth of over 80% at pH 3 and 0.3% oxgall (in comparison to control), were selected for identification and* in vitro* assays for probiotic properties. In the* in vitro* assays, a commercial probiotic* Lactobacillus* strain,* L. casei* Shirota, from Yakult fermented milk, was used as a reference strain.* Lactobacillus casei* Shirota was obtained from the culture collection of the Faculty of Pharmacy, Universiti Teknologi MARA, Malaysia.

### 2.2. Identification

Overnight culture (1.5 mL) of each of the nine LAB isolates in MRS broth was centrifuged at 5000 ×g for 10 min at room temperature. The cell pellet was used for extraction of total genomic DNA using the DNeasy Blood and Tissue Kit (Qiagen, Germany). For amplification of the 16S rRNA gene, universal primers F27 (5′-AGAGTTTGAT CMTGGCTCAG-3′) and R1492 (5′-TACGGYTACCTTGTTACGACTT-3′) were used [9, 10] with expected PCR product size of 1.5 kb. The PCR amplification was performed in 25 *μ*L reaction mixtures using a MyCycler Thermal Cycler (BioRad, USA). The PCR reaction mixture contained 2.5 *μ*L of PCR buffer (10X PCR amplification buffer containing 500 mM KCl, 100 mM Tris-HCl (pH 9.0), and 1% Triton X-100), 0.5 *μ*L of deoxynucleotide triphosphate (dNTP, i-DNA Biotechnology, Singapore) in concentration of 10 mM, 0.5 *μ*L of each primer in concentration of 10 *μ*M, 20 *μ*L of deionised water, 0.5 *μ*L of Taq DNA polymerase (Viogene, Taiwan, 2 U/*μ*L), and 0.5 *μ*L of template DNA (corresponding to approximately 50 to 100 ng of DNA). The PCR conditions were as follows: initial denaturation at 94°C for 4 min, 30 cycles of denaturation at 94°C for 1 min each, primer annealing at 55°C for 30 s and primer extension at 72°C for 2 min, and a final step of primer extension at 72°C for 5 min. The PCR product with expected size of 1.5 kb was excised from the gel and purified using MEGAquick-spin PCR and Agarose Gel DNA Extraction System (iNtRON Biotechnology, Korea). Each purified PCR product was ligated into PCR 2.1 TOPO vector using a TOPO TA cloning kit (Invitrogen, USA) and cloned into* E. coli* TOP 10 according to the manufacturer's instructions. The DNA sequence analysis was carried out for plasmid with the unique insert using an ABI 373XL automated sequencer (Applied Biosystems, USA) at both directions to obtain the full sequence of the amplicons. DNA sequence data sets were assembled using the Bioedit sequence alignment editor software, version 7.0.9.0 [11]. Sequence similarity values were determined using the basic local alignment search tool (BLAST) of the National Centre of Biotechnology Information (NCBI) [12]. Sequences with ≥97% similarity to the previously published sequences were used as the criteria to indicate species identity. Sequence alignment was carried out using CLUSTAL W program of the Bioedit software version 7. A phylogenetic tree was constructed based on the 16S rRNA gene sequence analysis where the analysis involved 51 nucleotide sequences comprising nine sequences of LAB obtained in this study, 41 sequences belonging to* Lactobacillus* species that were obtained from the GenBank and the sequence of* Lactococcus lactis *(AB100803.1) which was used as an outgroup. Evolutionary analyses were conducted using molecular evolutionary genetic analyses (MEGA) version 5. The evolutionary history was inferred using the neighbor-joining method. Bootstrapping was performed for 1000 replicates and the evolutionary distances were computed using the Tamura 3-parameter method [13]. Potential anomalous sequences of the 16S rRNA gene were examined by the Mallard [14] and the Bellerophon [15] programs. Nucleotide sequences of nine LAB isolates determined in this study were deposited in the GenBank (NCBI) database.

### 2.3. *In Vitro* Assessment of Characteristics for Survival in the Gastrointestinal Tract

#### 2.3.1. Acid Tolerance

The acid tolerance assay was tested according to Ehrmann et al. [16] with modifications. Cells of each LAB strain (in a final concentration of 7 to 8 log CFU/mL PBS) were inoculated (1%, v/v) into sterile PBS, adjusted to pH 3 with 1 N HCl (acidic condition) and PBS with normal pH 7.2 (control), and incubated anaerobically for 3 h at 37°C. After incubation, tenfold serial dilutions (up to 10^−7^) of each bacterial strain were prepared using PBS. Then 100 *μ*L of 10^−4^ to 10^−7^ dilutions from each sample was spread-plated on MRS agar and incubated anaerobically at 37°C for 24 h. After incubation, colonies on the plates were counted and enumerated as CFU/mL. Tolerance to acidic condition was estimated by comparing viable cell counts after exposure to acidic (pH 3) and normal (control) conditions. The assay was performed twice, each in triplicate.

#### 2.3.2. Bile Tolerance

The bile tolerance assay was tested according to Jacobsen et al. [17] with modifications. Overnight culture of each LAB strain, adjusted to a final concentration of 7 to 8 log CFU/mL, was inoculated (1%, v/v) into 10 mL of fresh MRS broth with or without (control) 0.3% (w/v) oxgall and incubated anaerobically at 37°C for 4 h, after which tenfold serial dilutions of up to 10^−7^ were prepared using PBS. Then 100 *μ*L of 10^−4^ to 10^−7^ dilutions from each sample was spread-plated on MRS agar and incubated anaerobically at 37°C for 24 h. After incubation, viability of bacterial cells was assessed by colony counts (CFU/mL) on the plates. Bile tolerance was estimated by comparing viable cell counts in MRS with and without bile (oxgall).

#### 2.3.3. Pancreatic Enzyme Tolerance

Tolerance to pancreatic enzymes was tested according to the method of Rönkä et al. [18] with modifications. Harvested cell pellet of overnight culture of each LAB strain was resuspended in PBS to a final concentration of 7 to 8 log CFU/mL and 1% (v/v) of resuspended cells was inoculated into 10 mL of the test solution (PBS containing 150 mM NaHCO_3_ and 1.9 mg/mL pancreatin (Sigma, USA), pH 8) and control solution (PBS, pH 7.2). The cultures were incubated anaerobically at 37°C for 3 h. After incubation, tenfold serial dilutions of up to 10^−7^ were prepared using PBS and 100 *μ*L of 10^−4^ to 10^−7^ dilutions from each sample was spread-plated on MRS agar. The plates were incubated anaerobically at 37°C for 24 h, after which viability of bacterial cells was assessed by colony counts (CFU/mL). Tolerance to pancreatic enzymes was estimated by comparing viable cell counts in test solution and control solution.

#### 2.3.4. Adherence Assay

The human intestinal epithelial cell line, Caco-2 cell line (ATCC 2102-CRL), was used in the adherence assay. The Caco-2 cells were routinely grown to 80–85% confluent in Dulbecco's Modified Eagle Medium (DMEM) (Sigma, USA) supplemented with 20% (v/v) fetal bovine serum (FBS) (Sigma, USA), 100 IU/mL penicillin (Sigma, USA), and 10 mg/mL streptomycin (Sigma, USA). The procedure used for the adherence assay followed that of Gopal et al. [19] with modifications. A cell suspension (1 × 10^5^ cell/mL DMEM) of Caco-2 cells was used for preparation of a monolayer of the cells on glass cover slips placed in six-well tissue culture plates. One mL of the cell suspension was added into each well of the plates containing fresh DMEM, and the plates were incubated overnight. Incubation for maintenance of cells and adherence assay was at 37°C in 5% CO_2_. For each LAB strain, cells from overnight culture (10 mL) were harvested by centrifugation at 4000 ×g for 10 min at 4°C, washed three times with sterile PBS (pH 7.2), and then resuspended in sterile PBS buffer (pH 7.2) to a final concentration of 8 log CFU/mL. Adherence assay was performed by adding 100 *μ*L of bacterial suspension onto the washed (once with PBS) monolayer of Caco-2 cells in the well containing 2 mL of fresh DMEM and incubated for 1 h at 37°C. After incubation, the monolayers were washed four times with PBS to remove unattached bacteria, then fixed with 3 mL of methanol, and incubated for 5 to 10 min at room temperature. The fixed monolayers were Gram stained and examined with a light microscope under oil immersion lens (Dialux, Leitz Wetzlar, Germany). Adherence was evaluated in 20 random microscopic fields and the number of adhered LAB cells per Caco-2 cell was determined. The assay was performed twice, each in triplicate.

### 2.4. Antibiotic Susceptibility Test (Minimum Inhibitory Concentration)

Antibiotic susceptibility test for the LAB strains was carried out using the broth microdilution method according to the ISO 10932/IDF 233 standard [20] with minor modifications. The antibiotics tested were ampicillin, clindamycin, gentamicin, streptomycin, tetracycline, erythromycin, kanamycin, and chloramphenicol (Sigma, USA). All the antibiotics were dissolved for preparing stock solutions of 1280 *μ*g/mL. The stock solutions were diluted in LAB susceptibility test medium (LSM) broth [21] to obtain solutions with concentrations of 0.25 to 128 *μ*g/mL. For preparation of bacterial inocula, colonies from overnight culture of each LAB strain were suspended in 5 mL 0.85% NaCl solution, adjusted to a turbidity of 0.2 ± 0.02 (OD_620 nm_), and diluted 1 : 500 in LSM broth. Then, 50 *μ*L of each diluted inoculum was added to each well of 96-well microdilution plates containing 50 *μ*L of an antibiotic solution, resulting in the concentration of about 5 log CFU/well for each bacterial inoculum. Inoculated plates were incubated anaerobically at 37°C for 48 h. After incubation, the minimum inhibitory concentration (MIC) values were determined as the lowest concentration of an antibiotic in which visible growth was inhibited and were compared with the MIC breakpoint values for heterofermentative lactobacilli recommended by the European Food Safety Authority (EFSA) Panel on Additives and Products or Substances used in Animal Feed [22]. Accuracy of the test was checked by parallel use of a quality control strain (*Enterococcus faecalis* ATCC 29212) as suggested by the Clinical and Laboratory Standards Institute (CLSI) [23]. The assay was performed twice, each in triplicate.

### 2.5. Antimicrobial Activity

#### 2.5.1. Antagonistic Activity against Pathogens

Twelve strains that are pathogenic to humans were used as test pathogens to investigate the antagonistic activity of the LAB strains. They were* Candida albicans* (ATCC 44831),* Enterococcus faecium* (ATCC 51558),* Staphylococcus epidermidis* (ATCC 12228),* Propionibacterium acnes* (ATCC 6919),* E. coli* (ATCC 29181),* Shigella sonnei* (ATCC 25931), and* Helicobacter pylori *(ATCC 43579) from the American type culture collection;* Enterobacter cloacae*,* Vibrio parahaemolyticus*, and* Listeria monocytogenes* were from the culture collection of Dr. Cheah Yoke Kqueen, Department of Biomedical Science, Faculty of Medicine and Health Science, Universiti Putra Malaysia;* Klebsiella pneumoniae *(K36) and* Staphylococcus aureus *(S244) were from the Institute of Medical Research, Malaysia.

The antagonistic activities of the LAB strains against the 12 pathogenic test strains were evaluated using the agar spot test described by Touré et al. [24] with modifications. Briefly, 2 *μ*L of overnight culture of each LAB strain (final concentration of 7 log CFU/mL) was spotted on MRS agar plates. The plates were dried for 30 min at room temperature and then incubated anaerobically at 37°C for 18 h in anaerobic jars (Oxoid, UK) containing gaspack (AnaeroGen, Oxoid, UK). After colony development, the plates were overlaid with 10 mL of soft (0.8% (w/v) agar) microorganism-specific medium, seeded with 1% (v/v) of an active overnight culture of the target pathogenic strain (final concentration of 7 log CFU/mL), and incubated aerobically at 37°C, except for* Candida albicans* where the incubation temperature was 24°C. The microorganism-specific media were yeast mold broth for* Candida albicans *(ATCC 44831), reinforced clostridial broth for* Propionibacterium acnes *(ATCC 6919), trypticase soy broth for* Staphylococcus aureus *(S244) and* Enterococcus faecium *(ATCC 51558), brain heart broth for* Listeria monocytogenes*, and nutrient broth for the other pathogenic strains (all media from Sigma, USA). After 48 h of incubation, measurements of inhibition zones around the LAB colonies were taken from the outer edge of the colonies to the outer edge of the clear zones. Inhibition zones of more than 20 mm, 10 to 20 mm, and less than 10 mm were considered as strong, intermediate, and low inhibitions, respectively. The test was performed twice, each in triplicate.

#### 2.5.2. Characterization of Antimicrobial Substances

The LAB strains were assayed for production of antimicrobial substances such as bacteriocin, hydrogen peroxide, and organic acids using the agar well diffusion technique described by Touré et al. [24] with modifications. The bacterial strains were grown in 25 mL of MRS broth at 37°C overnight, after which the cultures were centrifuged at 4000 ×g for 10 min at 4°C. The supernatant of each strain was divided into equal portions for different assays. For bacteriocin assay, the supernatant (5 mL) was treated with 1 mg/mL trypsin (Sigma, USA) or 1 mg/mL pronase (Sigma, USA). For organic acids assay, the supernatant (5 mL) was adjusted to pH 6.5 ± 0.1 using 1 N NaOH, and, for hydrogen peroxide assay, the supernatant (5 mL) was treated with 0.5 mg/mL catalase (Sigma, USA). Treated supernatants were filter sterilized through 0.22 *μ*m pore-size filters (Pall, USA), and 100 *μ*L was placed into wells (7 mm diameter) of MRS agar plates, overlaid with 10 mL of soft nutrient agar (Merck, Germany), and inoculated with 1% (v/v) of an overnight culture of* E. coli *(ATCC 29181)as test pathogen (indicator strain). The plates were kept at 4°C for 3 h for better diffusion of the treated supernatant and then incubated for 48 h at 37°C and diameters of inhibition zones (including the 7 mm well diameter) were measured. The assays were carried out twice, each in triplicate.

#### 2.5.3. Organic Acid Production Profile

Assays for organic acid production by the LAB strains were according to the method described by Erwin et al. [25] with modifications. Overnight culture (incubated at 37°C in anaerobic jar (Oxoid, UK) containing gaspack (AnaeroGen, Oxoid, UK, oxygen level <1%, CO_2_ level between 9 and 13%)) of each strain was centrifuged at 1500 ×g for 10 min at room temperature and 3 mL of the supernatant was added to 600 *μ*L of 24% metaphosphoric acid (in 3 N H_2_SO_4_). The mixture was kept at room temperature for 24 h and then centrifuged at 1500 ×g for 20 min at room temperature. For volatile fatty acids (acetic, propionic, isobutyric, butyric, isovaleric, valeric, and caproic acids) (VFA) determination, 0.5 mL of supernatant was added with 0.5 mL of 20 mM 4-methylvaleric acid, and 1 *μ*L of each sample was injected to a gas chromatograph (GC, Agilent Technologies, USA) with a flame ionization detector (FID) and fused-silica capillary column (30 m × 25 *μ*m, inside diameter). The temperature of column was set at 100 to 190°C with temperature programing at the rate of 5°C/min increments for optimal separation. Temperatures of oven, FID, and injector were 160, 250, and 230°C, respectively. Nitrogen with the flow rate of 1.0 mL/min was used as carrier gas. The internal standard was 20 mM 4-methylvaleric acid. Acetic acid (20 mM), propionic acid (10 mM), butyric acid (10 mM), isobutyric acid (10 mM), valeric acid (10 mM), isovaleric acid (10 mM), and 4-methylvaleric acid (10 mM) were used as standard solutions to identify the peaks. For nonvolatile fatty acids (lactic and succinic acids) (non-VFA) determination, 0.5 mL of supernatant was added with 0.5 mL of 20 mM fumaric acid and 1 *μ*L of each sample was injected to the GC. Temperatures of oven, FID, and injector were 180, 150, and 110°C, respectively. Nitrogen with the flow rate of 1.0 mL/min was used as carrier gas. The internal standard was 20 mM fumaric acid. Lactic acid (20 mM), succinic acid (10 mM), and fumaric acid (10 mM) were used as standard solutions to identify the peaks. The assays were carried out twice, each in triplicate.

### 2.6. Statistical Analysis

Data of each assay were analyzed by one-way analysis of variance (ANOVA) using the SAS (Statistical Analysis System, 2008) program version 9.2. Comparison among treatment means was performed using Duncan's new multiple range test. Differences were considered significant at *P* < 0.05.

## 3. Results

### 3.1. Identification Using 16S rRNA Gene Sequences

The results of comparative 16S rRNA gene analysis showed that all nine LAB isolates belonged to the genus* Lactobacillus *(Table 1). Of the three isolates from human milk, one isolate (HM1) was 99% similar to* L. acidophilus* while the other two isolates (HM2 and HM3) were 99% similar to* L. fermentum*. One isolate (FG1) from fermented grapes and two isolates (FD1 and FD2) from fermented dates were 99% similar to* L. buchneri*. The three isolates (BF1, BF2, and BF3) from infant feces were 99% similar to* L. casei*. The 16S rRNA gene sequences of the nine* Lactobacillus* strains were deposited in the GenBank database under the accession numbers JN188382 to JN188390 for isolates HM1, HM2, HM3, FG1, FD1, FD2, BF1, BF2, and BF3, respectively. The pure cultures of the nine* Lactobacillus* strains were deposited in the Microbial Culture Collection Unit (UNiCC) of Universiti Putra Malaysia under the accession numbers UPMC 999 to UPMC 1007 for isolates HM1, HM2, HM3, FG1, FD1, FD2, BF1, BF2, and BF3, respectively.

### 3.2. Phylogenetic Analysis Based on 16S rRNA Gene

**Figure 1:**
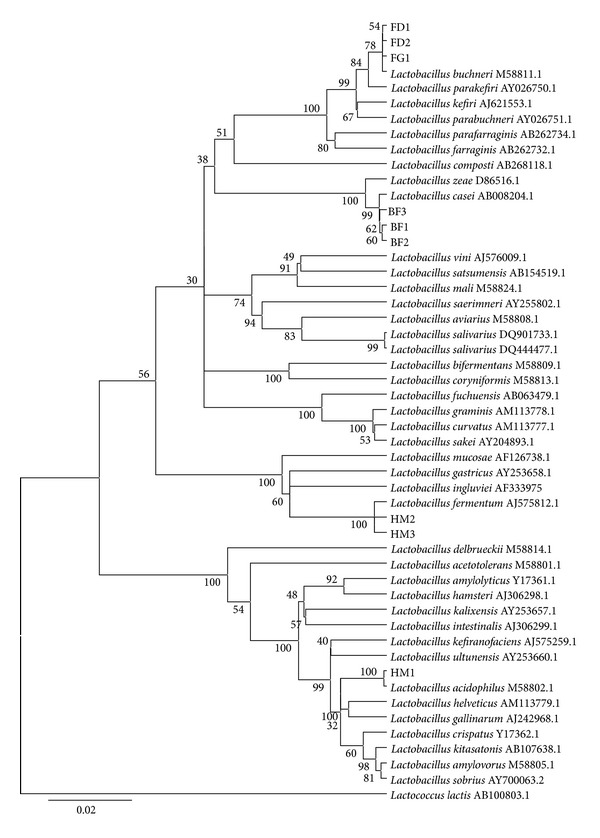
Phylogenetic tree based on the neighbor-joining method of 16S rRNA gene sequences. The analysis involved nine sequences of* Lactobacillus* strains obtained in this study, 41 sequences of* Lactobacillus* species obtained from the GenBank, and the outgroup was* Lactococcus lactis *AB100803.1. Bootstrap values above 50% are indicated at the nodes of the tree. The scale bar represents 0.02-nucleotide substitutes per position.

Figure 1 shows the phylogenetic tree based on 16S rRNA gene sequence analysis, depicting the phylogenetic relationships among the nine* Lactobacillus* strains and 41* Lactobacillus* type strains obtained from the GenBank.* Lactococcus lactis *(AB100803.1) was used as the outgroup. Strains FD1 and FD2 isolated from fermented dates and FG1 isolated from fermented grapes were clustered together and were monophyletic with* L. buchneri *M58811.1 (bootstrap value of 78%). The three strains, BF1, BF2, and BF3, isolated from infant feces, were grouped together and formed a monophyletic clade with* L. casei *AB008204.1 with a bootstrap value of 99%. Two strains, HM2 and HM3, isolated from human milk, were monophyletic with* L. fermentum *AJ575812.1 with a remarkable bootstrap value of 100% and the other strain, HM1, also isolated from human milk, was monophyletic with* L. acidophilus *M58802.1, with a bootstrap value of 100%.

### 3.3. *In Vitro* Assessment of Characteristics for Survival in the Gastrointestinal Tract

#### 3.3.1. Acid, Bile, and Pancreatic Enzymes Tolerance

Table 2 shows the viability of the nine* Lactobacillus* strains isolated in this study and the reference strain* L. casei* Shirota at pH 3 and pH 7.2 (control). All nine* Lactobacillus* strains showed good tolerance to acid (pH 3), but the level of tolerance varied among the strains. Of the nine* Lactobacillus* strains, eight (*L. acidophilus* HM1,* L. fermentum* HM2 and HM3,* L. buchneri* FG1, FD1, and FD2, and* L. casei* BF1 and BF2) showed high acid tolerance with loss in cell viability of only 0.0 to 0.18 log units. The acid tolerance levels of these* Lactobacillus* strains were significantly (*P* < 0.05) higher than those of* L. casei* BF3 with cell viability loss of 0.34 log unit and the reference strain* L. casei* Shirota with loss in cell viability of 0.37 log unit.

The results of bile tolerance for all nine* Lactobacillus* strains and the reference strain are shown in Table 3. All nine* Lactobacillus* strains and the reference strain exhibited tolerance to 0.3% bile (oxgall). However, the degrees of tolerance varied among the strains.* Lactobacillus acidophilus* HM1,* L. buchneri* FG1, FD1, and FD2, and the reference strain* L. casei *Shirota showed the highest (*P* < 0.05) tolerance to bile salt, with an increase in cell viability of 0.01 and 0.02 log units exhibited by* L. acidophilus* HM1 and* L. buchneri* FD2, respectively, and a slight reduction in cell viability of 0.01 to 0.04 log units showed by* L. casei *Shirota and* L. buchneri* FG1 and FD1. The other* Lactobacillus* strains exhibited lower levels of tolerance to bile salt, with reduction in cell viability of 0.45 to 0.76 log units.

All nine* Lactobacillus* strains and the reference strain exhibited good tolerance to pancreatic enzymes (Table 4).* Lactobacillus buchneri* FD2 was not affected at all by pancreatic enzymes, with no reduction in cell viability. Other strains such as* L. acidophilus* HM1,* L. buchneri* FG1 and FD1,* L. casei* BF2, and the reference strain* L. casei *Shirota showed high tolerance to pancreatic enzymes with reduction in cell viability of only 0.07 to 0.10 log units. The two* L. fermentum* strains (HM2 and HM3) and two* L. casei* strains (BF1 and BF3) showed lower (*P* < 0.05) tolerance to the pancreatic enzymes with the reduction in cell viability of 0.15 to 0.27 log units.

#### 3.3.2. Adherence Ability

The adherence abilities of the* Lactobacillus* strains are shown in Table 5. All nine* Lactobacillus* strains and the reference strain were able to adhere to Caco-2 cells, but the adherence ability varied widely among the strains. The highest (*P* < 0.05) adherence ability was exhibited by* L. fermentum* HM3 isolated from human milk, with 37.7 attached cells/Caco-2 cell, and the lowest (*P* < 0.05) adherence ability was shown by* L. casei *BF1, with 13.7 attached cells/Caco-2 cell.* Lactobacillus buchneri *FD2 and FG1,* L. acidophilus* HM1,* L. fermentum* HM2, and* L. buchneri* FD1 with 35.9, 34.8, 33.5, 32.4, and 30.3 attached cells/Caco-2 cell, respectively, also showed high attachment ability. The other two* Lactobacillus* strains (*L. casei* BF2 and BF3) and the reference strain* L. casei *Shirota had moderate adherence ability of 15.8 to 19.7 attached cells/Caco-2 cell.

### 3.4. Antibiotic Susceptibility Test

The results of MIC values for antibiotic susceptibility of the* Lactobacillus* strains against eight tested antibiotics are shown in Table 6. All nine* Lactobacillus* strains and the reference strain exhibited MIC values lower than the MIC breakpoint values recommended for heterofermentative* Lactobacillus* strains by EFSA [22] for all the tested antibiotics, namely, ampicillin, gentamicin, kanamycin, streptomycin, erythromycin, clindamycin, tetracycline, and chloramphenicol. Vancomycin was not tested since all nine* Lactobacillus* strains and the reference strain were heterofermentative* Lactobacillus* strains, as, according to the EFSA [22], susceptibility testing of heterofermentative* Lactobacillus* strains against vancomycin is not required. In addition, since none of the strains was resistant to the tested antibiotics, no further studies on their antibiotic resistance are needed according to EFSA [22].

### 3.5. Antimicrobial Activity

#### 3.5.1. Antagonistic Effects

The results of antagonistic effects of the* Lactobacillus* strains against 12 pathogenic strains are shown in Table 7. All nine* Lactobacillus* strains and the reference strain showed antagonistic effects against all pathogenic strains tested, but the degrees of antagonism varied among the* Lactobacillus* strains. The results showed that all the isolated* Lactobacillus* strains, except* L. acidophilus* HM1, exhibited strong inhibition on the growth of* Staphylococcus epidermidis* (ATCC12228),* Enterobacter cloacae*, and* Listeria monocytogenes* (inhibition zones of more than 20 mm), and the three* L. casei* strains (BF1, BF2, and BF3) showed strong antagonistic activities against* Helicobacter pylori* (inhibition zones of more than 20 mm) and good inhibition against* Staphylococcus aureus* (inhibition zones of 19 to 20 mm). However, most of the* Lactobacillus* strains (including the reference strain) showed low inhibitory activities against* Klebsiella pneumonia* (K36) (inhibition zones of less than 10 mm). Among the nine isolated* Lactobacillus* strains,* L. casei* BF1 was the most effective strain in inhibiting the growth of the test pathogens. It showed the highest (*P* < 0.05) inhibitory actions against 8 of 12 test pathogens. In contrast,* L. acidophilus* HM1 was the least (*P* < 0.05) effective strain, showing the lowest inhibitory activities against 11 of 12 test pathogens. Overall, many of the isolated* Lactobacillus* strains showed better (*P* < 0.05) antagonistic activities against the test pathogens than the reference strain* L. casei* Shirota.

#### 3.5.2. Characterization of Inhibitory Substances

The antimicrobial substance produced by the nine isolated* Lactobacillus* strains was characterized by the agar well diffusion assay against an indicator strain,* E. coli *(ATCC 29181). The results showed that culture supernatants of all nine isolated* Lactobacillus* strains and the reference strain treated with pronase (1 mg/mL) or trypsin (1 mg/mL) did not affect their inhibitory activities against the indicator strain (Table 8). This indicated that inhibitory effects of the* Lactobacillus* strains were not due to bacteriocin production. Culture supernatants treated with catalase also did not affect the inhibitory activities of the* Lactobacillus* strains against the indicator strain. This showed that inhibition by the* Lactobacillus* strains was not due to hydrogen peroxide production. However, neutralized supernatants (pH 6.5) of all* Lactobacillus* strains did not have any inhibitory activity against the indicator strain, which indicated that inhibitory effects of the* Lactobacillus* strains were due to their organic acid productions (Table 8).

#### 3.5.3. Profile of Organic Acid Production

The organic acid production profiles of the* Lactobacillus* strains are presented in Table 9. Lactic acid was the most abundant organic acid produced by all the* Lactobacillus* strains, followed by acetic acid. The amounts of lactic acid produced varied among the strains, ranging from the lowest (*P* < 0.05) amount of 143.65 mM produced by* L. acidophilus* HM1 to the highest amount of 356.95 mM produced by* L. casei* BF1. Generally, all three strains of* L. casei* (BF1, BF2, and BF3) produced very high amounts of lactic acid (310.97 to 356.95 mM), while* L. fermentum* HM2 and HM3 and* L. buchneri* FD1 and FD2 produced more moderate amounts of 205.70 to 227.07 mM. Production of acetic acid also varied among the* Lactobacillus* strains. The highest (*P* < 0.05) amounts of acetic acid were produced by* L. fermentum* HM2 and HM3 and* L. buchneri* FG1 (with 130.14, 125.71, and 124.16 mM, resp.). The rest of the strains produced acetic acid ranging from 68.08 to 92.85 mM. Succinic acid production varied very widely among the* Lactobacillus* strains.* Lactobacillus acidophilus* HM1,* L. fermentum* HM2 and HM3, and* L. buchneri* FG1 produced more than 20 mM of succinic acid, but the rest of the* Lactobacillus* strains produced about 1 to 2 mM. Other acids such as propionic, isobutyric, butyric, isovaleric, and caproic acids either were produced in trace amounts or were not produced by the* Lactobacillus* strains.

## 4. Discussion

In this study, nine* Lactobacillus* strains isolated from human milk (*L. acidophilus* HM1 and* L. fermentum* HM2 and HM3), infant feces (*L. casei* BF1, BF2, and BF3), fermented grapes (*L. buchneri* FG1), and fermented dates (*L. buchneri* FD1 and FD2) were evaluated for their potential probiotic characteristics and antimicrobial activity against some human pathogens.

Every potential probiotic strain is expected to tolerate the condition of the GIT in order to be able to provide its beneficial effect on the host. The ability to tolerate acid, bile, and pancreatic enzymes and to adhere to the intestinal epithelial cells has been considered as good indicator for the survival of a bacterial strain in the GIT, and these characteristics are often assessed* in vitro* in the preliminary selection of a probiotic strain. Although* in vitro* assessments may not be able to totally mimic the actual* in situ* conditions in the gut ecosystem, they remain powerful tools for rapid screening of potential strains. They permit an enormous level of simplification of the system under study, allowing a large number of strains to be investigated for a specific probiotic property. The use of* in vivo* studies for initial investigation of probiotic properties of new potential probiotic strains is not only time-consuming but also expensive. Thus, the use of* in vitro* assays to assess and select the most effective strain for* in vivo* investigations is a more logical option [16, 26]. Dunne et al. [2] reported that adoption of proper criteria for the* in vitro* selection of probiotic bacteria can result in the isolation of strains capable of performing effectively in the GIT.

In the present study, pH 3 was used to investigate the acid tolerance of the* Lactobacillus* strains as pH 3 has been considered as a standard pH for investigation of acid tolerance of probiotic strains in many studies [27–30]. The results showed that all nine isolated* Lactobacillus* strains exhibited good acid tolerance at pH 3 for 3 h, with eight strains showing significantly better acid tolerance than the reference strain* L. casei* Shirota. Ehrmann et al. [16] also reported that strains of* L. reuteri*,* L. salivarius*, and* L. animalis* were able to tolerate pH 3 for 4 h, but the degrees of tolerance varied among the strains. Earlier, Charteris et al. [31] have pointed out in their review that most* Lactobacillus *spp. are able to tolerate pH 4 for 1 hour, but the percentage of cell viability varies considerably among different strains. It is also apparent from the results of the current study that acid tolerance of the* Lactobacillus* strains was not related to the source of isolation as the level of acid tolerance could vary considerably among the strains from the same source. For example, among the three* L. casei* strains isolated from infant feces,* L. casei* BF3 exhibited significantly lower acid tolerance than* L. casei* BF1 and BF2, and, between the two* L. buchneri* strains isolated from fermented dates,* L. buchneri* FD2 showed significantly higher acid tolerance than* L. buchneri* FD1.

The ability to tolerate bile salt at a concentration of 0.3% has a physiological significance because it is a level normally encountered in human intestine [32]. Gilliland et al. [33] have also reported that the normal concentration of bile salt in human small intestine is 0.3% (w/v), but some studies have suggested that bile concentration is variable and unpredictable, changing according to diet composition and in a close relationship with the secretion of pancreatic enzymes [34, 35]. However, in many studies, the standard level of 0.3% bile was considered for investigation of bile tolerance of potential probiotic* Lactobacillus* strains [17, 28, 30, 33, 36, 37]. Thus, in the present study, 0.3% bile concentration was used. All nine isolated* Lactobacillus* strains showed good bile tolerance at this concentration of bile salt. Similar results were reported by Kõll et al. [30] in which all 67* Lactobacillus* strains tested exhibited tolerance at 0.3% bile. Jin et al. [38] also found that all 12* Lactobacillus* strains tested were able to tolerate 0.3% of bile salt, while Jacobsen et al. [17] reported that 41 of 42 tested* Lactobacillus* strains could tolerate bile at this concentration.

In the current study, the degree of bile tolerance varied considerably with the strains. It was strain-specific and was not influenced by the environment of the isolation source. Kõll et al. [30] also found that the ability to tolerate bile salt was strain-specific among the tested* Lactobacillus* strains. Recently, Sahadeva et al. [28] also reported that* L. acidophilus*,* L. casei *Shirota,* Streptococcus thermophilus*, and* Bifidobacterium* from four brands of commercially cultured milk drinks showed a strain-specific profile of bile tolerance at 0.3% of bile salt.

Pancreatic enzymes are secreted into the small intestine through the pancreatic duct and they are involved in digestion of proteins, carbohydrates, and fats in foods. As such, some studies have included the ability to tolerate the presence of pancreatic enzymes as another criterion for selection of probiotic cultures [18, 39]. In this study, 3 h of exposure to pancreatic enzymes had little adverse effect on the survival of the nine isolated* Lactobacillus* strains. All nine strains showed good tolerance to pancreatic enzymes, with slight variations in the degree of tolerance among the strains. Similar results were reported by Rönkä et al. [18] who found that 3 h of incubation in growth medium containing pancreatic enzymes had little effect on the viability of* L. brevis* strains. Ruiz-Moyano et al. [37] also reported that 46 out of 51 tested LAB strains survived after 3 h of treating with 1.9 mg/mL of pancreatic enzymes.

In addition to its ability to survive the stressful gastric environment of the GIT, every potential probiotic strain is also expected to be able to attach to the epithelial cells of the intestine in order to colonize and be established in the intestine [40]. Furthermore, high adherence to the intestine is necessary for releasing some probiotic bioeffects, for example, cholesterol lowering effects, immune-modulation, and antimicrobial activities against pathogens. Caco-2 cell line, which was used in this study, is a human intestinal cell line that has been extensively used as a cellular model for assessing attachment of bacteria because it has morphological and physiological characteristics of normal human enterocytes [41, 42]. In the present study, adherence of the nine isolated* Lactobacillus* strains to the Caco-2 cell line was in range of 14 to 38 cells per Caco-2 cell. The adherence ability varied among the strains, indicating that it is strain-specific. Similar findings were reported by Jacobsen et al. [17] who studied 47* Lactobacillus *strains for their ability to adhere to Caco-2 cells and found considerable variations, from strong to low adhesion, among the strains. The strain-specific adhesion of* Lactobacillus *strains on different epithelial cell lines has been well documented by Del Piano et al. [43]. Gopal et al. [19] also found that* L. rhamnosus *DR20,* L. acidophilus *HN017, and* B. lactis *DR10 exhibited strong ability to adhere to the Caco-2 and HT29 human epithelial cell lines. Some studies indicated that exopolysaccharides on the cell walls are adhesion molecules which can affect the adherence ability of* Lactobacillus* strains [44, 45]. It was also found that some adhesin factors of* Lactobacillus* strains are proteins that are loosely bound to surface components of epithelial cells by noncovalent interaction, such as electrostatic interaction [46].

According to EFSA [22], for the assessment of susceptibility of bacterial strains to antibiotics, serial twofold dilution methods should be used and relevant quality control strains should be included. In the dilution methods, the MIC is defined as the lowest concentration of the antibiotic that inhibits bacterial growth. In the present study, a serial twofold broth microdilution method was used to assess the susceptibility of the nine* Lactobacillus* strains to eight antibiotics suggested by EFSA [22] for heterofermentative* Lactobacillus* strains. The results showed that none of the nine* Lactobacillus* strains was resistant to any of the tested antibiotics. The results of antibiotic susceptibility of the quality control strain (*Enterococcus faecalis *ATCC 29212) were in the range suggested by CLSI [23], indicating the accuracy of the susceptibility testing.

Recently, Carasi et al. [47] examined six strains of* L. kefiri* for their susceptibility against eight antibiotics using the broth microdilution method and reported that all the strains were susceptible to tetracycline, clindamycin, streptomycin, ampicillin, erythromycin, kanamycin, and gentamicin, but two strains were resistant to chloramphenicol and were further studied for their chloramphenicol resistance gene. Mayrhofer et al. [48] also tested the susceptibility of 101 strains of the* L. acidophilus* group against 13 antibiotics using the broth microdilution method. They found narrow and broad unimodal and bimodal MIC distributions in the* L. acidophilus* group for the tested antimicrobial agents. Besides the microdilution method, diffusion methods such as Etest and disk diffusion have been used to assess the susceptibility of bacterial strains to antibiotics. Mayrhofer et al. [49] compared the results of the broth microdilution, disk diffusion, and Etest methods of 104 strains of the* L. acidophilus *group and reported that the MIC values obtained from the broth microdilution and the Etest methods were generally similar and they correlated with the inhibition zone diameters determined with the disk diffusion method. Korhonen et al. [50] also investigated 75 strains of* L. rhamnosus* for their susceptibility to six antibiotics using the agar dilution, broth microdilution, and Etest methods. They found that most of the tested strains were susceptible to ampicillin, clindamycin, erythromycin, gentamicin, streptomycin, and tetracycline, but three strains were resistant to clindamycin, erythromycin, and streptomycin, and one strain was resistant to streptomycin and tetracycline. Although qualitative or semiqualitative methods, such as the diffusion methods, are commonly used to determine MIC indirectly, at present, they are generally not acceptable by EFSA [22].

Antimicrobial activity against pathogens is another important attribute to be considered in the selection of potential probiotic strains for maintaining a healthy microbial balance in the GIT. In the present study, all nine isolated* Lactobacillus* strains showed antagonistic activity against all the 12 test pathogens, which are pathogenic to humans. Many of the* Lactobacillus* strains showed higher antagonistic effects against the test pathogens than the reference strain* L. casei* Shirota. In particular, the inhibitory effects of* L. casei* BF1, BF2, and BF3 on* H. pylori *and* S. aureus* were significantly higher than those of* L. casei* Shirota.* Helicobacter pylori* infection of the stomach can cause chronic gastritis, gastric or duodenal ulcers, and gastric cancer. Treatment of* H. pylori* infection using multiple antibiotic regimens may not eradicate it effectively and reinfection may occur. New treatment strategies such as using probiotic strains to reduce the growth of* H. pylori* in humans have been considered. Cats et al. [51] had reported that* L. casei* Shirota, which was used as a reference strain in the present study, was capable of inhibiting the growth of* H. pylori in vitro*, and* in vivo* (in human subjects), there was a slight, but nonsignificant, trend towards an inhibitory effect of* L. casei* Shirota on* H. pylori*. The three* L. casei* strains (BF1, BF2, and BF3) which showed significantly higher inhibitory effect than* L. casei* Shirota in the current study should be further studied, as they may be more promising biotherapeutic agents for* H. pylori* infection than* L. casei* Shirota. Similarly, further investigation on* L. casei* BF1, BF2, and BF3 should be conducted to explore their potential as biotherapeutic agents for* S. aureus *infection. It is well known that* S. aureus *has become resistant to multiple antibiotics, and new therapeutic agents are required.

The concept of antagonistic activity of LAB against pathogenic strains has been well documented in a review by Šušković et al. [52]. The antagonistic activity has mostly been attributed to the production of antimicrobial substances or metabolites such as organic acids, hydrogen peroxide, ethanol, diacetyl, acetaldehyde, acetoin, carbon dioxide, reuterin, reutericyclin, and bacteriocins by the probiotic strains. This activity, together with the mechanism of competitive exclusion, in which probiotic strains compete with pathogens for nutrients and attachment sites, would prevent colonization of the intestine by pathogens [53]. Among the antimicrobial substances, organic acids (especially lactic and acetic acids), hydrogen peroxide, and bacteriocins are the most common antimicrobial substances that have been reported to be produced by probiotic strains. In the current study, the antagonistic activities of all nine isolated* Lactobacillus* strains were found to be due to their organic acid production not hydrogen peroxide or bacteriocin production. Jin [54] had also found that the inhibitory effects of 12* Lactobacillus* strains on pathogenic* Salmonella* and* E. coli* were due to their production of organic acids. Recently, Neal-McKinney et al. [55] reported that the antagonistic activities of* L. acidophilus*,* L. crispatus*,* L. gallinarum*, and* L. helveticus* against six strains of* Campylobacter jejuni* were due to organic acid production not bacteriocin production.

The organic acid profiles of the nine* Lactobacillus* strains in the present study showed that lactic acid was the predominant acid produced by all the strains, followed by acetic acid, and succinic acid was produced in much lesser amount. The other acids, such as propionic, butyric, isobutyric, valeric, and isovaleric acids, were either not produced or produced in trace amounts by some of the strains. It has been reported that lactic and acetic acids are the main organic acids involved in antimicrobial activity of* Lactobacillus* strains [56]. The antifungal activities of lactic and acetic acids produced by lactobacilli against* Aspergilli* and* Fusarium* were investigated by Zalan et al. [57] and they reported that none of the investigated* Aspergilli* was inhibited, but the inhibitory effect of the acids against* Fusarium* increased with increasing concentrations of acids. They also reported that the production of organic acids varied between species and also between strains of the same subspecies of 10 tested strains of* L. casei*,* L. rhamnosus*,* L. plantarum*,* L. paracasei*, and* L. curvatus*. Species- and strain-specific antimicrobial activities of lactobacilli have also been reported by Corsetti et al. [58] who tested 232 strains from nine species of* Lactobacillus* isolated from sourdoughs for their antagonistic activity against sourdough-related microorganisms. Of these, 77 strains showed antagonistic activity with a clear species- and strain-specific profile. Similarly, the results of the present study showed that the antagonistic activities and production of organic acids varied among the nine* Lactobacillus* strains and were strain-specific. The effectiveness of the* Lactobacillus* strain in inhibiting the test pathogens generally corresponded with the amounts of organic acids produced, particularly lactic acid. For instance,* L. casei* BF1, which was the most effective strain in inhibiting the growth of the test pathogens, produced more than twice the amount of lactic acid when compared to* L. acidophilus* HM1, which was the least effective strain.

## 5. Conclusions

The results of this* in vitro* study indicated that all nine* Lactobacillus* strains were able to survive in the GIT and attached to the epithelial cells, while none of them was antibiotic resistant. Since all nine* Lactobacillus* strains showed strong antagonistic activities against a wide range of pathogens to humans, they could be considered as good potential probiotic candidates for treatment and prevention of infections. They should be studied further as biotherapeutic agents for treatments of specific disease conditions. The strains should also be investigated further for other probiotic bioactivities that have human health benefits.

## Figures and Tables

**Table 1:** Identification of the nine lactic acid bacterial isolates using 16S rRNA gene sequences.

Isolate	Source	Accession number of isolate	The nearest matched species from GenBank	Similarity (%)
HM1	Human milk	JN188382	*L. acidophilus *	99
HM2	Human milk	JN188383	*L. fermentum *	99
HM3	Human milk	JN188384	*L. fermentum *	99
FG1	Fermented grapes	JN188385	*L. buchneri *	99
FD1	Fermented dates	JN188386	*L. buchneri *	99
FD2	Fermented dates	JN188387	*L. buchneri *	99
BF1	Infant feces	JN188388	*L. casei *	99
BF3	Infant feces	JN188389	*L. casei *	99
BF2	Infant feces	JN188390	*L. casei *	99

Similarity values were determined using the basic local alignment search tool (BLAST) of the GenBank. Sequences with ≥97% similarity to the previously published sequences were used as the criteria to indicate species identity.

**Table 2:** Viability of *Lactobacillus* strains (log⁡CFU/mL) after 3 h exposure to pH 3 and pH 7.2 (control).

*Lactobacillus* strain	Cell viability (log⁡CFU/mL)^1^		Reduction in cell viability (log units)^1^
	pH 7.2	pH 3	
*L. casei *Shirota∗	7.11 ± 0.07	6.74 ± 0.06	0.37^a^
*L. acidophilus* HM1	7.22 ± 0.07	7.15 ± 0.06	0.07^bc^
*L. fermentum* HM2	7.30 ± 0.03	7.16 ± 0.05	0.14^bc^
*L. fermentum* HM3	7.85 ± 0.07	7.69 ± 0.10	0.16^bc^
*L. buchneri* FG1	6.97 ± 0.03	6.83 ± 0.05	0.14^bc^
*L. buchneri* FD1	7.43 ± 0.05	7.25 ± 0.03	0.18^b^
*L. buchneri* FD2	7.16 ± 0.06	7.16 ± 0.04	0.00^c^
*L. casei* BF1	7.30 ± 0.03	7.27 ± 0.03	0.03^bc^
*L. casei* BF2	7.48 ± 0.08	7.46 ± 0.07	0.02^bc^
*L. casei* BF3	7.12 ± 0.05	6.78 ± 0.07	0.34^a^

^1^Values are means ± SD of two independent experiments, each with triplicate.

^a–c^Means within a column with different superscripts are significantly different (*P* < 0.05).

∗Reference strain.

**Table 3:** Growth of *Lactobacillus* strains in MRS broth (control) and MRS broth containing 0.3% bile salt.

*Lactobacillus* strain	Cell viability (log⁡CFU/mL)^1^		Reduction (−)/increase (+) in cell viability (log units)^1^
	MRS	MRS + 0.3% bile salt	
*L. casei *Shirota∗	8.75 ± 0.13	8.71 ± 0.12	−0.04^a^
*L. acidophilus* HM1	7.40 ± 0.31	7.41 ± 0.31	+0.01^a^
*L. fermentum* HM2	8.73 ± 0.03	8.04 ± 0.04	−0.69^cd^
*L. fermentum* HM3	8.85 ± 0.01	8.17 ± 0.05	−0.68^cd^
*L. buchneri* FG1	7.59 ± 0.05	7.56 ± 0.07	−0.03^a^
*L. buchneri* FD1	7.53 ± 0.05	7.55 ± 0.09	+0.02^a^
*L. buchneri* FD2	7.63 ± 0.05	7.62 ± 0.11	−0.01^a^
*L. casei* BF1	8.25 ± 0.02	7.49 ± 0.07	−0.76^d^
*L. casei* BF2	8.16 ± 0.16	7.71 ± 0.06	−0.45^b^
*L. casei* BF3	8.08 ± 0.16	7.52 ± 0.08	−0.56^bc^

^1^Values are means ± SD of two independent experiments, each with triplicate.

^a–d^Means within a column with different superscripts are significantly different (*P* < 0.05).

∗Reference strain.

**Table 4:** Viability of *Lactobacillus* strains (log⁡CFU/mL) after 3 h in PBS with and without (control) 1.9 mg/mL pancreatic enzymes.

*Lactobacillus* strain	Cell viability (log⁡CFU/mL)^1^		Reduction in cell viability (log units)^1^
	Control	1.9 mg/mL pancreatic enzymes	
*L. casei *Shirota∗	7.81 ± 0.09	7.72 ± 0.07	0.09^bc^
*L. acidophilus* HM1	7.45 ± 0.05	7.33 ± 0.07	0.12^bc^
*L. fermentum* HM2	7.77 ± 0.05	7.50 ± 0.08	0.27^a^
*L. fermentum* HM3	7.97 ± 0.04	7.70 ± 0.08	0.27^a^
*L. buchneri* FG1	7.00 ± 0.04	6.90 ± 0.04	0.10^bc^
*L. buchneri* FD1	7.35 ± 0.05	7.28 ± 0.07	0.07^bc^
*L. buchneri* FD2	7.74 ± 0.09	7.74 ± 0.03	0.00^c^
*L. casei* BF1	7.88 ± 0.04	7.67 ± 0.07	0.21^ab^
*L. casei* BF2	7.28 ± 0.11	7.19 ± 0.09	0.09^bc^
*L. casei* BF3	7.76 ± 0.07	7.61 ± 0.09	0.15^ab^

^1^Values are means ± SD of two independent experiments, each with triplicate.

^a–c^Means within a column with different superscripts are significantly different (*P* < 0.05).

∗Reference strain.

**Table 5:** Adherence of cells of *Lactobacillus* strains to Caco-2 cell.

*Lactobacillus* strain	Adherence index (*Lactobacillus* cells per Caco-2 cell)^1^
*L. casei *Shirota∗	19.7 ± 0.3^g^
*L. acidophilus* HM1	33.5 ± 0.9^d^
*L. fermentum* HM2	32.4 ± 0.4^e^
*L. fermentum* HM3	37.7 ± 0.6^a^
*L. buchneri* FG1	34.8 ± 0.5^c^
*L. buchneri* FD1	30.3 ± 0.2^f^
*L. buchneri* FD2	35.9 ± 0.7^b^
*L. casei* BF1	13.7 ± 0.3^j^
*L. casei* BF2	18.5 ± 0.3^h^
*L. casei* BF3	15.8 ± 0.4^i^

^1^Values are means ± SD of two independent experiments, each in triplicate.

Adherence was evaluated in 20 random microscopic fields.

^a–j^Means with different superscripts are significantly different (*P* < 0.05).

∗Reference strain.

**Table 6:** Minimum inhibitory concentrations (MIC) for antibiotic susceptibility of *Lactobacillus* strains.

	Antibiotic [MIC (*μ*g/mL)]							
Strain	Ampicillin	Gentamicin	Kanamycin	Streptomycin	Erythromycin	Clindamycin	Tetracycline	Chloramphenicol
Breakpoint∗	4	16	64	64	1	1	8	4
*L. casei *Shirota∗∗	<0.25	<8	<64	<32	<1	<0.063	<1	<4
*L. acidophilus* HM1	<0.063	<0.125	<0.125	<0.5	<0.063	<0.063	<0.063	<0.063
*L. fermentum* HM2	<0.063	<4	<8	<8	<0.5	<0.063	<1	<1
*L. fermentum* HM3	<0.063	<4	<8	<4	<0.5	<0.063	<1	<1
*L. buchneri* FG1	<0.5	<2	<8	<8	<0.25	<0.063	<2	<1
*L. buchneri* FD1	<0.5	<4	<8	<8	<0.25	<0.063	<2	<1
*L. buchneri* FD2	<0.5	<2	<8	<2	<0.125	<0.063	<2	<1
*L. casei* BF1	<0.125	<8	<8	<4	<0.125	<0.063	<0.5	<0.25
*L. casei* BF2	<0.5	<4	<8	<4	<0.125	<0.25	<1	<0.5
*L. casei* BF3	<0.5	<8	<8	<4	<0.125	<0.125	<1	<0.5

*Values are provided by EFSA [22] for facultative heterofermentative *Lactobacillus* strains; according to EFSA, susceptibility testing of heterofermentative *Lactobacillus* strains against vancomycin is not required.

**Reference strain.

**Table 7:** Antagonistic activity of *Lactobacillus* strains against test pathogens.

*Lactobacillus* strain	Inhibition zone (from outer edge of *Lactobacillus* colony to outer edge of clear zone) (mm)^1^											
	*Candida albicans* (ATCC 44831)	*Propionibacterium acnes* (ATCC 6919)	*Shigella sonnei* (ATCC 25931)	*Helicobacter pylori* (ATCC 43579)	*Enterobacter cloacae *	*Vibrio parahaemolyticus *	*Listeria monocytogenes *	*E. coli* (ATCC 29181)	*Enterococcus faecium* (ATCC 51558)	*Staphylococcus epidermidis* (ATCC12228)	*Staphylococcus aureus* (S244)	*Klebsiella pneumoniae* (K36)
*L. casei *Shirota∗	13.8^bc^	11.2^c^	13.5^d^	14.2^e^	18.7^d^	6.5^e^	14.0^f^	11.7^c^	11.8^b^	18.3^b^	14.0^d^	7.8^d^
*L. acidophilus* HM1	9.7^d^	3.3^e^	6.3^e^	16.2^d^	7.2^e^	7.0^e^	13.7^f^	11.8^c^	11.2^bc^	12.0^c^	6.7^e^	5.0^e^
*L. fermentum* HM2	13.5^c^	11.2^c^	14.5^cd^	18.7^c^	20.8^c^	11.7^cd^	24.0^b^	14.7^ab^	13.7^a^	22.5^a^	14.5^d^	10.5^b^
*L. fermentum* HM3	15.2^a^	11.2^c^	14.5^cd^	19.3^c^	20.8^c^	11.7^cd^	25.7^a^	14.5^ab^	14.2^a^	23.3^a^	14.5^d^	10.5^b^
*L. buchneri* FG1	15.0^ab^	4.3^de^	15.0^bc^	15.5^d^	22.5^ab^	12.3^bc^	22.3^cd^	12.0^c^	11.2^bc^	23.3^a^	17.0^c^	11.0^b^
*L. buchneri* FD1	15.0^ab^	5.2^d^	14.3^cd^	15.3^d^	22.5^ab^	12.2^cd^	23.8^bc^	11.3^c^	10.7^c^	24.2^a^	17.5^c^	11.2^b^
*L. buchneri* FD2	14.7^ab^	4.3^de^	13.7^d^	16.3^d^	23.2^a^	11.2^c^	21.8^d^	11.5^c^	10.5^c^	24.2^a^	17.2^c^	12.7^a^
*L. casei* BF1∗∗	15.0^ab^	16.5^a^	16.0^ab^	20.7^b^	21.5^bc^	14.5^a^	20.7^de^	14.8^a^	11.8^b^	23.7^a^	20.2^a^	9.5^c^
*L. casei* BF2∗∗	15.5^a^	16.5^a^	16.7^a^	22.2^a^	22.3^abc^	13.3^b^	19.7^e^	14.0^ab^	11.7^b^	23.0^a^	19.2^ab^	8.8^c^
*L. casei* BF3∗∗	15.0^ab^	14.8^b^	16.5^a^	22.0^a^	22.2^abc^	14.5^a^	20.8^de^	13.8^b^	11.3^bc^	23.0^a^	18.8^b^	9.0^c^
SEM	0.19	0.52	0.32	0.47	0.59	0.30	0.42	0.17	0.14	0.39	0.40	0.25

^1^Values are means of two independent experiments, each in triplicate.

^a–f^Means with different superscripts within a column are significantly different (*P* < 0.05).

*Reference strain.

**These strains that showed higher antagonistic activities than other strains against most of tested pathogenic strains, also produced higher amounts of lactic acid (Table 9).

**Table 8:** Inhibitory activity of treated and untreated supernatants of *Lactobacillus* strains against *E. coli *(ATCC 29181) as indicator strain.

*Lactobacillus* strain	Diameter of inhibition zone (mm) including 7 mm well diameter^1^				
	Untreated supernatant (control)	Neutralized supernatant (pH 6.5)	Supernatant + pronase (1 mg/mL)	Supernatant + trypsin (1 mg/mL)	Supernatant + catalase (0.5 mg/mL)
*L. casei *Shirota∗	16.9 ± 0.8	—	17.9 ± 0.6	17.1 ± 0.0	15.9 ± 0.2
*L. acidophilus* HM1	15.1 ± 0.9	—	14.5 ± 2.1	15.3 ± 0.9	14.8 ± 0.2
*L. fermentum* HM2	16.4 ± 1.4	—	15.7 ± 1.4	15.0 ± 0.5	17.0 ± 1.9
*L. fermentum* HM3	15.7 ± 1.0	—	15.5 ± 0.7	15.8 ± 2.1	15.5 ± 1.6
*L. buchneri* FG1	16.3 ± 1.0	—	16.8 ± 0.2	15.2 ± 0.7	16.5 ± 1.2
*L. buchneri* FD1	15.5 ± 0.4	—	16.0 ± 0.5	15.8 ± 0.7	15.2 ± 0.2
*L. buchneri* FD2	15.9 ± 0.8	—	17.2 ± 1.2	15.3 ± 0.9	15.3 ± 1.4
*L. casei* BF1	18.1 ± 1.2	—	17.8 ± 0.2	19.3 ± 0.5	17.7 ± 0.5
*L. casei* BF2	18.2 ± 0.7	—	18.0 ± 0.9	18.8 ± 0.7	17.8 ± 0.2
*L. casei* BF3	17.9 ± 0.9	—	18.5 ± 0.2	17.7 ± 0.5	17.8 ± 0.7

^1^Values are means ± SD of two independent experiments, each in triplicate.

— No inhibition.

∗Reference strain.

**Table 9:** Organic acid production of *Lactobacillus* strains.

*Lactobacillus* strain	Non-volatile fatty acids (mM)^1^			Volatile fatty acids (mM)^1^					
	Lactic acid	Succinic acid	Acetic acid	Propionic acid	Isobutyric acid	Butyric acid	Isovaleric acid	Valeric acid	Caproic acid
*L. casei *Shirota∗	185.78 ± 10.85^f^	1.41 ± 0.13^d^	68.08 ± 2.63^c^	ND	ND	ND	ND	ND	ND
*L. acidophilus* HM1	143.65 ± 5.38^g^	25.18 ± 0.40^b^	71.54 ± 3.09^c^	0.32 ± 0.01	ND	ND	ND	ND	ND
*L. fermentum* HM2	227.07 ± 12.75^c^	23.97 ± 0.41^c^	130.14 ± 7.89^a^	ND	ND	ND	ND	ND	ND
*L. fermentum* HM3	209.80 ± 4.52^de^	24.00 ± 0.56^c^	125.71 ± 5.35^a^	ND	ND	ND	ND	ND	0.39 ± 0.01
*L. buchneri* FG1	196.08 ± 11.36^ef^	26.33 ± 0.83^a^	124.16 ± 7.38^a^	ND	ND	ND	0.22 ± 0.00	ND	ND
*L. buchneri* FD1	205.70 ± 3.65^de^	1.78 ± 0.15^d^	82.46 ± 4.49^b^	0.15 ± 0.03	ND	ND	0.29 ± 0.00	ND	ND
*L. buchneri* FD2	218.70 ± 3.65^cd^	2.08 ± 0.57^d^	92.85 ± 5.29^b^	0.33 ± 0.02	0.26 ± 0.05	0.38 ± 0.03	0.25 ± 0.05	0.16 ± 0.03	0.16 ± 0.03
*L. casei* BF1∗∗	356.95 ± 5.14^a^	1.77 ± 0.17^d^	70.76 ± 11.19^c^	ND	0.15 ± 0.01	ND	0.25 ± 0.04	0.16 ± 0.01	0.64 ± 0.02
*L. casei* BF2∗∗	353.15 ± 13.23^a^	1.73 ± 0.17^d^	81.18 ± 13.88^bc^	ND	ND	ND	0.27 ± 0.01	0.17 ± 0.02	ND
*L. casei* BF3∗∗	310.97 ± 3.50^b^	1.71 ± 0.28^d^	70.89 ± 4.90^c^	0.35 ± 0.03	0.22 ± 0.01	0.38 ± 0.02	0.21 ± 0.02	0.34 ± 0.00	0.12 ± 0.01

^1^Values are means ± SD of two independent experiments, each in triplicate.

^a–f^Means within a column with different superscripts are significantly different (*P* < 0.05).

ND: not detected.

∗Reference strain.

∗∗These strains, which produced higher amounts of lactic acid than other strains, generally also exhibited higher antagonistic activities against most of the tested pathogenic strains (Table 7).
